# Evaluation of the NG-TEST^®^ blood culture PREP kit for ESBL directly from positive blood cultures using the NG-TEST^®^ CTX-M MULTI lateral flow immunoassay

**DOI:** 10.1093/jacamr/dlag080

**Published:** 2026-05-19

**Authors:** Saoussen Oueslati, Niroshika Visvanathan, Giulia Orlandi, Blandine Baumy, Laura Francius, Hervé Volland, Arnaud Chalin, Thierry Naas

**Affiliations:** Team ‘Resist’, UMR1184 ‘Infectious Disease Models and Innovative Therapies (IDMIT)’ INSERM, Université Paris-Saclay, CEA, Healthi, Le Kremlin-Bicêtre, France; Department of Bacteriology-Hygiene, Bicêtre Hospital, APHP Paris-Saclay, Le Kremlin-Bicêtre, France; Essonne (Île-de-France), SEPSIS Comprehensive Center – IHU SEPSIS, Gif-sur-Yvette, France; Team ‘Resist’, UMR1184 ‘Infectious Disease Models and Innovative Therapies (IDMIT)’ INSERM, Université Paris-Saclay, CEA, Healthi, Le Kremlin-Bicêtre, France; Team ‘Resist’, UMR1184 ‘Infectious Disease Models and Innovative Therapies (IDMIT)’ INSERM, Université Paris-Saclay, CEA, Healthi, Le Kremlin-Bicêtre, France; Research and Development Department, NG Biotech, Guipry, France; Research and Development Department, NG Biotech, Guipry, France; Département Médicaments et Technologies Pour la Santé, Université Paris-Saclay, CEA, INRAE, Gif-sur-Yvette 91191, France; Research and Development Department, NG Biotech, Guipry, France; Team ‘Resist’, UMR1184 ‘Infectious Disease Models and Innovative Therapies (IDMIT)’ INSERM, Université Paris-Saclay, CEA, Healthi, Le Kremlin-Bicêtre, France; Department of Bacteriology-Hygiene, Bicêtre Hospital, APHP Paris-Saclay, Le Kremlin-Bicêtre, France; Essonne (Île-de-France), SEPSIS Comprehensive Center – IHU SEPSIS, Gif-sur-Yvette, France

## Abstract

**Background:**

ESBL-producing Enterobacterales are a major cause of bloodstream infections, requiring rapid detection for timely antimicrobial therapy. We evaluated the performance of the NG-TEST^®^ CTX-M MULTI lateral flow immunoassay combined with the NG-TEST^®^ Blood Culture Prep (BCP) Kit for detecting CTX-M ESBLs directly from positive blood cultures.

**Methods:**

In total 165 clinical Enterobacterales were used to spike blood culture bottles. These included 32 non–CTX-M producers, 120 CTX-M producers belonging to the five main CTX-M groups, and 13 *Kluyvera* sp. Blood cultures were placed in the BACT/ALERT^®^ VIRTUO^®^ system (bioMérieux); upon bacterial growth detection by the system a UriSelect 4 Agar (Bio-Rad) was inoculated to verify purity. Bacteria were prepared using the NG-TEST^®^ BCP Kit followed by the NG-TEST^®^ CTX-M MULTI.

**Results:**

The NG-TEST^®^ CTX-M MULTI detected all 120 CTX-M–producing isolates expressing CTX-M variants belonging to the five groups (1, 2, 8, 9 and 25) from both colonies and blood cultures, resulting in 100% sensitivity. The NG-TEST^®^ BCP Kit removed all the patients’ blood cells and reliably extracted bacteria irrespective of the bacterial species. Non–CTX-M producers gave negative results, confirming 100% specificity. Among CTX-M–producing *Kluyvera* species, eight isolates tested positive, while five were repeatedly negative, and consistent with prior reports.

**Conclusions:**

Our results demonstrate that the combination of the NG-TEST^®^ BCP Kit and the NG-TEST^®^ CTX-M MULTI systems provides a rapid (<20 min), user-friendly, robust and accurate tool for species-independent detection of CTX-M–type ESBLs directly from positive blood cultures, supporting earlier targeted antimicrobial therapy in bloodstream infections.

## Introduction

Bloodstream infections (BSIs) are severe bacterial diseases associated with high morbidity and mortality. Rapid identification of the pathogen and its resistance profile is crucial, especially in sepsis, where delays in appropriate therapy increase mortality.^1^ Among Gram-negative bacteria, ESBL-producing Enterobacterales are a major concern due to limited treatment options, often restricted to last-resort antibiotics.^2^ Infections caused by ESBL-producing *Escherichia coli* and *Klebsiella pneumoniae* are linked to higher treatment failure, longer hospital stays and increased mortality.^3,4^ CTX-M enzymes now account for most ESBL resistance worldwide.^2,5^ In BSIs, inappropriate empirical therapy is more frequent with ESBL-producing pathogens and is associated with worse outcomes.^6^ Conventional identification and susceptibility testing require 48–72 h after blood culture positivity, delaying targeted treatment and prolonging broad-spectrum antibiotic use.^7^

Rapid diagnostic tools (RDTs) applied directly to positive blood cultures have emerged as a key strategy to address these delays and improve clinical outcomes.^7–10^ These include MS, molecular assays, WGS and rapid phenotypic testing.^7–10^ These technologies have significantly improved the management of BSIs, especially with CTX-M–type ESBL-producing organisms, by shortening time to pathogen identification and antimicrobial resistance detection.^9–11^ Although effective, many RTDs require costly equipment and specialized personnel.^10,12^ Biochemical tests are simple alternatives that have proven useful for predicting the hydrolysis of β-lactam substrates in bacterial colonies.^7,13^ Although they cannot be applied directly to positive blood cultures, they can be performed on short subcultures (3–4 h) derived from positive blood cultures.^13^

Lateral flow immunoassays (LFIAs) provide a rapid, low-complexity alternative for ESBL detection. These assays detect β-lactamase enzymes directly from bacterial pellets obtained from positive blood cultures or short subcultures, with results available in 15–30 min.^8,14,15^ LFIAs targeting CTX-M enzymes have demonstrated high sensitivity and specificity and are particularly well suited for laboratories lacking molecular infrastructure.^8,14,15^ These tests are inexpensive, easy to perform, and particularly useful in low-resource settings, but are limited to specific resistance enzymes.

Efficient bacterial extraction from positive blood cultures is essential to enable these rapid methods. Various extraction methods allow separation of bacterial cells from blood components within minutes to a few hours.^14–17^ These approaches reduce human cell debris and inhibitory substances, yielding bacterial pellets suitable for downstream applications, including LFIA.

This study aimed to evaluate the performance of the NG-TEST^®^ Blood Culture PREP (BCP) kit for the rapid preparation of bacterial pellets from positive blood cultures, enabling direct testing with the NG-TEST^®^ CTX-M MULTI assay for detection of CTX-M groups 1, 2, 8, 9 and 25.

## Material and methods

### Bacterial strains

A total of 165 clinical Enterobacterales isolates were included in this study. They corresponded to *E. coli* (*n* = 70), *K. pneumoniae* (*n* = 34), *Klebsiella oxytoca* (*n* = 8), *Klebsiella aerogenes* (*n* = 3), *Proteus mirabilis* (*n* = 3), *Enterobacter cloacae* complex (*n* = 17), *Citrobacter freundii* (*n* = 14), *Citrobacter koseri* (*n* = 2), *Citrobacter braakii* (*n* = 1) and *Kluyvera* spp. (*n* = 13). Most of these isolates were from a previous study, and their resistome was characterized by WGS.^14^

Included in this study were 120 Enterobacterales expressing 40 different CTX-M variants belonging to the five CTX-M subgroups (Table 1). Compared with a previous study by Bernabeu *et al.*,^14^ 14 novel variants were tested (Table 1).

**Table 1.** NG-TEST^®^ CTX-M MULTI test results on colonies and on positive blood cultures using the NG-TEST^®^ BCP kit

Bacterial species (*n*)	β-Lactamase content^a^	CTX-M group (no. of variants tested/total no. of variants in BLDB)	No. of strain(s)	NG-TEST CTX-M MULTI result on colonies	NG-TEST CTX-M-MULTI result on blood cultures
Acquired CTX-M variants					
* Citrobacter freundii*, *Citrobacter koseri*, *Escherichia coli* (6)	CTX-M-1	**G1 (21/137)**	8	+	+
* Enterobacter cloacae* complex, *E. coli*	CTX-M-3		2	+	+
* E. coli*, *Klebsiella pneumoniae*	CTX-M-10		2	+	+
* C. freundii* (3), *E. cloacae* complex (11), *E. coli* (17), *K. pneumoniae* (13), *Klebsiella oxytoca* (3), *Klebsiella aerogenes* (1), *Proteus mirabilis* (1)	CTX-M-15		49	+	+
* C. freundii*, *E. coli*	CTX-M-32		2	+	+
*K. pneumoniae*	CTX-M-33		1	+	+
*C. freundii*	CTX-M-36		1	+	+
*E. coli*	CTX-M-37		1	+	+
*C. freundii*	CTX-M-55		1	+	+
*E. coli*	CTX-M-57		1	+	+
*E. coli*, *P. mirabilis*	CTX-M-71		2	+	+
*E. coli*	CTX-M-82		1	+	+
*E. coli* (2)	CTX-M-101		2	+	+
*K. pneumoniae*	CTX-M-127		1	+	+
*C. freundii*	CTX-M-139		1	+	+
*E. coli* (2)	CTX-M-182		2	+	+
*Citrobacter braakii*	CTX-M-194		1	+	+
*C. freundii*	CTX-M-216		1	+	+
*C. freundii*	CTX-M-224		1	+	+
*E. cloacae* complex	CTX-M-232		1	+	+
*C. freundii*	CTX-M-245		1	+	+
*E. coli* (2), *K. pneumoniae*	CTX-M-2	**G2 (1/31)**	3	+	+
*K. pneumoniae* (2)	CTX-M-25	**G25 (5/14)**	2	+	+
*K. pneumoniae*	CTX-M-26		1	+	+
*E. cloacae* complex, *E. coli* (3), *K. pneumoniae* (2)	CTX-M-39		6	+	+
*E. coli*	CTX-M-94		1	+	+
*E. coli*	CTX-M-100		1	+	+
*E. coli*, *K. pneumoniae*	CTX-M-8	**G8 (2/3)**	2	+	+
*C. freundii*	CTX-M-40		1	+	+
*E. cloacae* complex, *E. coli* (2)	CTX-M-9	**G9 (11/80)**	3	+	+
*E. coli*	CTX-M-13		1	+	+
*C. freundii*, *E. coli* (7)	CTX-M-14		8	+	+
*E. coli*	CTX-M-17		1	+	+
*E. coli*	CTX-M-18		1	+	+
*K. pneumoniae*	CTX-M-19		1	+	+
*E. coli*	CTX-M-24		1	+	+
*E. coli* (3)	CTX-M-27		3	+	+
*K. pneumoniae*	CTX-M-65		1	+	+
*E. coli* (2)	CTX-M-93		2	+	+
*C. koseri*	CTX-M-196		1	+	+
Non-CTXM-ESBLs and non-ESBLs					
*C. freundii*, *E. coli* (5), *K. oxytoca*, *K. pneumoniae* (3)	SHV-ESBL		10	−	−
*K. pneumoniae*, *E. coli*, *K. oxytoca* (2)	TEM-1		4	−	−
*E. coli* (4), *K. aerogenes* (2), *K. pneumoniae* (2)	TEM-BLSE		8	−	−
*E. coli*, *K. pneumoniae* (2), *P. mirabilis*, *E. cloacae* complex	VEB-like		5	−	−
*K. oxytoca*, *K. pneumoniae*	GES-like		2	−	−
*K. oxytoca*	CMY-2		1	−	−
*E. coli*	WT		1	−	−
*E. cloacae* complex	WT		1	−	−
Natural and chromosome-encoded CTX-Ms					
*Kluyvera ascorbata*	CTX-M-like		6	+	+
*K. ascorbata*	CTX-M-like		2	−	−
*Kluyvera cochlae*	CTX-M-like		2	−	−
*Kluyvera cryocrescens*	CTX-M-like		2	+	+
*Kluyvera georgiana*	CTX-M-like		1	−	−
Total number of tested isolates			165		

BLDB, Beta-Lactamase Database.

^a^Bold denotes novel variants that were not included in the previous evaluation.^14^

Thirty-two non–CTX-M–producing Enterobacterales were used as negative controls to assess specificity. Among these strains, 25 were non–CTX-M ESBL producers such as SHV, TEM, VEB and GES, and 7 were WT or non-ESBL producers (Table 1). A selection of 13 *Kluyvera* sp. strains known to possess chromosomal *bla*_CTX-M_-like genes, which are mostly weakly or not expressed, were also tested.

### Blood culture spiking

**Figure 1.**
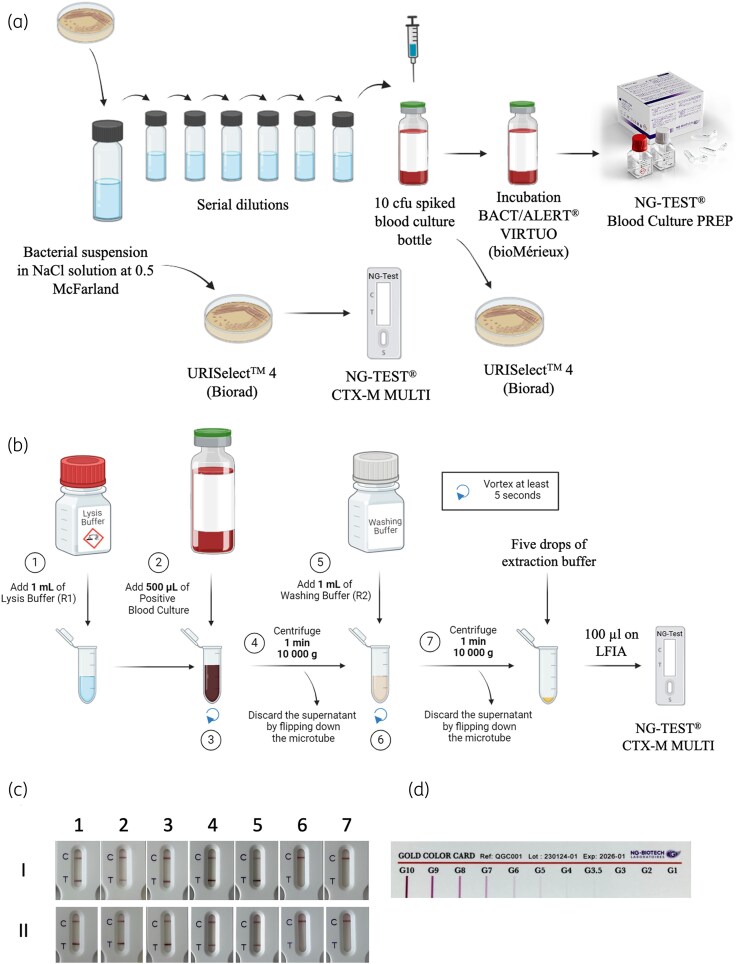
Experimental procedure. (a) Blood culture spiking. (b) NG-TEST^®^ BCP protocol. (c) Example of NG-TEST CTX-M-MULTI results from (I) positive blood cultures and (II) colonies grown on UriSelect 4 Agar plates (Bio-Rad) of (1) CTX-M-1–producing *Escherichia coli*; (2) CTX-M-9–producing *Enterobacter cloacae*; (3) CTX-M-8–producing *E. coli*; (4) CTX-M-2–producing *E. coli*; (5) CTX-M-25–producing *Klebsiella pneumoniae*; (6) VEB-1–producing *E. cloacae*; (7) SHV-5–producing *K. pneumoniae.* (d) Intensity ruler from NG-Biotech. After 15 min migration, the intensity of each band is compared by eye with those of the intensity ruler and scored accordingly. C, control line; T, test line. Created with BioRender.com.

The strains were isolated on UriSelect 4 Agar chromogenic medium (Bio-Rad, Marnes-la-Coquette, France) to verify their purity. A 0.5 McFarland suspension was prepared to inoculate a Mueller–Hinton (MH) agar plate (Bio-Rad) and blood culture bottles (BACT/ALERT^®^ FA Plus; bioMérieux, Marcy l’Étoile, France) containing 8–10 mL of blood with 1–10 cfu. (Figure 1a). Time to positivity was recorded, and purity was confirmed on UriSelect 4 Agar. Using the NG-TEST^®^ BCP kit, 500 µL of blood culture was used for bacterial extraction (Figure 1a).

### NG-TEST^®^ BCP

Positive blood cultures were processed according to the manufacturer's protocol (NG Biotech; Figure 1b). Briefly, 0.5 mL of blood culture was mixed with 1 mL of lysis buffer, vortexed for 5 s, and centrifuged for 1 min at 10 000 × **g** at room temperature (∼20°C). The supernatant was discarded, and the pellet was washed with 1 mL of washing buffer, followed by a second 1 min centrifugation. After the supernatant was discarded, the pellet was resuspended with five drops of the NG-TEST^®^ CTX-M MULTI extraction buffer, vortexed for 5 s, and 100 µL was applied to the LFIA strip.

### NG-TEST^®^ CTX-M MULTI assay

Three isolated colonies grown on MH plates were touched with a 1 µL loop and resuspended in 150 µL of extraction buffer, and vortexed for 5 s. A 100 µL aliquot was then applied to the LFIA strip, following the manufacturer’s instructions (NG Biotech). Test results from both colony and blood culture extracts were visually read after 15 min by observing the appearance of a red test line alongside the internal control (Figure 1c). Readings were independently performed by two blinded observers, and results were recorded using the intensity scale shown in Figure 1d.

### Statistical analysis

The 95% CI, average and SDs were calculated using the online MedCalc statistical software (https://www.medcalc.org/calc/diagnostic_test.php).

## Results

The results were compiled into a comparative table of NG-TEST^®^ CTX-M MULTI outcomes obtained from isolated colonies and positive blood cultures with the NG TEST^®^ BCP kit (Table 1). All spiked blood cultures were flagged as positive by the BACT/ALERT^®^ VIRTUO^®^ automated system after 11.5 ± 3 h.

All 26 variants tested in the Bernabeu *et al.* study,^14^ as well as the 14 new variants (CTX-M-25, 26, 33, 36, 39, 40, 101, 139, 194, 196, 216, 224, 232 and 245), were equally well detected on colonies and from blood cultures. NG-TEST^®^ CTX-M MULTI unambiguously detected all 120 CTX-M–producing isolates, irrespective of the bacterial species they belonged to (Table 1). The Enterobacterales isolates expressing non–CTX-M ESBLs (*n* = 25) as well as the non-ESBL isolates (*n* = 7) yielded negative results, confirming the high specificity of the NG-TEST^®^ CTX-M MULTI, whether tested on colonies or on bacteria extracted with the NG-TEST^®^ BCP assay.

Finally, five *Kluyvera* spp. isolates (two *K. ascorbata*, one *K. georgiana* and two *K. cochleae*) consistently tested negative, both on colonies and on extracted bacterial samples (Table 1). These findings are consistent with those of Bernabeu *et al.*,^14^ who reported that CTX-M–like enzymes in some *Kluyvera* isolates are expressed at levels too low to be detected.

Our study showed that the NG-TEST^®^ CTX-M MULTI detected all Enterobacterales with acquired CTX-M production (120/120), extracted from blood cultures with the NG TEST^®^ BCP kit without any false positives (0/32). Thus, on our panel of isolates the sensitivity was 100% (95% CI 96.9%–100%) and the specificity 100% (95% CI 83.9%–100%). As shown in Figure 1c, the NG-TEST^®^ BCP effectively removed all RBCs, resulting in strips without red staining and yielding results comparable to those obtained from isolated colonies. After 15 min of migration the intensity of the bands was always between 7 and 10, compared with the intensity ruler, thus allowing unambiguous band interpretation (Figure 1c).

## Discussion

By bypassing overnight subculture, rapid extraction shortens time to pathogen identification and resistance detection, enabling earlier optimization of antimicrobial therapy in sepsis and other BSIs.^10^ Commercial blood culture preparation kits, such as the Sepsityper^®^ kit (Bruker, Hamburg, Germany), Vitek^®^ MS Blood Culture Kit (bioMérieux) and Qvella FAST System (Qvella Inc., Richmond Hill, Canada), as well as kits compatible with molecular assays and WGS, rapidly process positive cultures by separating bacteria from blood components, allowing direct identification of bacteria and/or resistance testing within 20–60 min.^9,10^ However, these methods require specialized equipment and trained personnel, and are expensive. Home-made assays are alternatives but are often time-consuming and labour intensive. In contrast, the NG-TEST BCP assay is rapid (under 5 min), easy, standardized, compliant with European Regulations for In Vitro Diagnostic Medical Devices, and requires minimal hands-on-time, thus improving laboratory efficiency and facilitating the integration of rapid diagnostic tools into routine clinical microbiology practice.

ESBLs, especially CTX-M-type, are the main source of cephalosporin resistance in Enterobacterales of human origin in France and in many other European countries.^2,5,13,14^ The NG-TEST^®^ CTX-M MULTI LFIA has demonstrated excellent biological performance and is currently the only commercially available assay capable of detecting all five CTX-M groups.^14,18^ Combining it with the NG-TEST^®^ BCP did not impair its excellent performance because all the previously detected CTX-M variants were detected, including 14 novel variants belonging to different CTX-M-groups and representing the most prevalent variants. This combination of assays is well adapted to the French, and to a larger extent the global, epidemiology of CTX-M producers. The entire process for a single blood culture required less than 20 min, including 5 min for the NG-TEST^®^ BCP procedure, as indicated in the manufacturer’s protocol, and 15 min for migration on the strip; however, in most cases, the band appeared in under 1 min in case of positivity, as previously shown.^14^

Routine use of the NG-TEST^®^ BCP procedure for ESBL detection in positive blood cultures will require a diagnostic stewardship programme that considers patient risk profiles as well as the local prevalence of CTX-M, other ESBLs, plasmid-encoded cephalosporinases (pAmpC) and carbapenemases. Such an approach should improve antimicrobial management by enabling early initiation of carbapenem therapy when CTX-M is detected in settings with low carbapenemase prevalence, while also helping to reduce unnecessary carbapenem use. This is particularly relevant in countries such as France, where CTX-M enzymes account for the vast majority of ESBLs and the prevalence of pAmpC and carbapenemases remains low.^14^ As carbapenemase producers often also produce CTX-M enzymes, in regions with a high prevalence of carbapenemases, combining the NG-TEST^®^ CTX-M MULTI with an LFIA targeting the five main carbapenemases, such as NG-TEST^®^ CARBA 5, would enable discrimination between isolates producing CTX-M alone and those co-producing CTX-M and carbapenemases.

Although our results are promising, they were obtained with spiked blood cultures, thus clinical usefulness needs to be further evaluated on real clinical blood cultures with larger numbers of CTX-M–negative isolates including non-fermenters. In addition, more CTX-M variants need to be tested; although those that were tested correspond to most prevalent variants worldwide,^5^ they represent only 13% (39/283) of the known variants listed in the Beta-Lactamase Database (http://bldb.eu).^19^ Finally, our results should be further confirmed in countries where the prevalence and the epidemiology of ESBL producers might be different from France and where pAmpC (DHA, CMY) are more prevalent.

### Conclusions

LFIAs are valuable confirmatory tests for detecting antibiotic resistance, particularly β-lactamases in Gram-negative bacteria,^8^ and meet the WHO ASSURED criteria for ideal point-of-care tests: Affordable, Sensitive, Specific, User-friendly, Rapid and robust, Equipment-free, and Deliverable.^20^ Like the NG-TEST^®^ CTX-M MULTI, the NG-TEST^®^ BCP assay can be stored and used at room temperature, making it suitable for laboratories with limited resources. While their combined use is simple and robust, it still requires a benchtop centrifuge and electricity, which may be limiting in resource-constrained settings. In these settings, the BL-DetecTool represents an attractive alternative, as it is a stand-alone system based on a simple and rapid filtration–concentration device that can be implemented in virtually any microbiology laboratory without the need for additional equipment, electricity or highly trained personnel.^15^

The clinical benefit of rapid diagnostic tools is greatest when their use is integrated with active antimicrobial stewardship programmes. Multiple studies have demonstrated that the combination of rapid resistance detection and stewardship intervention significantly reduces time to appropriate therapy, shortens hospital length of stay, decreases carbapenem use, and improves survival in patients with BSIs caused by ESBL-producing organisms.^11,21^ This integrated approach is especially critical in regions with high ESBL endemicity, where delayed or inappropriate therapy is common and therapeutic options may be limited.
